# Demographic history of an elusive carnivore: using museums to inform management

**DOI:** 10.1111/j.1752-4571.2012.00241.x

**Published:** 2012-02-07

**Authors:** Joseph D Holbrook, Randy W DeYoung, Michael E Tewes, John H Young

**Affiliations:** 1 Caesar Kleberg Wildlife Research Institute, MSC 218, Texas A&M University–KingsvilleKingsville, TX, USA; 2 Texas Parks and Wildlife DepartmentAustin, TX, USA

**Keywords:** effective population size, genetic differentiation, genetic diversity, microsatellite DNA loci, *Puma concolor*, Texas

## Abstract

Elusive carnivores present a challenge to managers because traditional survey methods are not suitable. We applied a genetic approach using museum specimens to examine how historical and recent conditions influenced the demographic history of *Puma concolor* in western and southern Texas, USA. We used 10 microsatellite loci and indexed population trends by estimating historical and recent genetic diversity, genetic differentiation and effective population size. Mountain lions in southern Texas exhibited a 9% decline in genetic diversity, whereas diversity remained stable in western Texas. Genetic differentiation between western and southern Texas was minimal historically (*F*_ST_ = 0.04, *P* < 0.01), but increased 2–2.5 times in our recent sample. An index of genetic drift for southern Texas was seven to eight times that of western Texas, presumably contributing to the current differentiation between western and southern Texas. Furthermore, southern Texas exhibited a *>*50% temporal decline in effective population size, whereas western Texas showed no change. Our results illustrate that population declines and genetic drift have occurred in southern Texas, likely because of contemporary habitat loss and predator control. Population monitoring may be needed to ensure the persistence of mountain lions in the southern Texas region. This study highlights the utility of sampling museum collections to examine demographic histories and inform wildlife management.

## Introduction

Natural resource conservation and management relies on surveys or other data to monitor population trends (Marsh and Trenham 2007). Changes in census size and demographic parameters can inform harvest prescriptions, justify management intervention and highlight overall conservation status (Witmer 2005). Population trends of large carnivores are of particular interest because of the biological integrity carnivores provide to ecosystems (Estes et al. 2011). Large carnivores are generally territorial and elusive and inhabit dense or rugged habitats (Witmer 2005). As a result, survey and monitoring programs that rely on traditional techniques, such as marking individuals, can be logistically and financially demanding (Barea-Azcon et al. 2007).

Genetic tools can assist the monitoring of carnivore populations (e.g. De Barbra et al. 2010). Genetic data can discriminate individuals or species and thus provide estimates of abundance and vital rates and characterize changes in geographical distribution (Schwartz et al. 2006). The use of genetic data has become relatively common to investigate the abundance and distribution of many species of carnivores (Boulanger et al. 2004; McKelvey et al. 2006). Furthermore, genetic techniques can be applied to assess demographic trends through time (Wandeler et al. 2007). Comparisons of genetic data from museum specimens and contemporary samples can elucidate the effects of historical and recent events on evolutionary processes, such as gene flow and genetic drift. Genetic analysis of historical samples has informed the conservation and management of *Ursus arctos* (L.) (brown bear; Miller and Waits 2003), *Puma concolor coryi* (B.) (Florida panther; Culver et al. 2008) and *Canis lupus* (L.) (gray wolf; Flagstad et al. 2003).

Throughout North America, *Puma concolor* (L.) (mountain lion) has experienced severe declines in census size and geographical distribution because of habitat loss and predator management policies (Logan and Sweanor 2001; Anderson et al. 2010). With the exception of the Florida panther, breeding populations of mountain lions occur in the western half of the continent. During the mid–late 1900s, most western states in the United States regulated the harvest of mountain lions (Anderson et al. 2010). Regulation allowed populations in some areas to recover to historical levels (Logan and Sweanor 2001). Today, large populations generally exhibit moderate levels of genetic diversity and low genetic differentiation (Culver et al. 2000; Anderson et al. 2004). Small and peripheral populations generally exhibit lower diversity and high differentiation, presumably because of reduced opportunities for gene flow (i.e. central-marginal hypothesis; Eckert et al. 2008) and habitat loss (Ernest et al. 2003).

**Figure 1:**
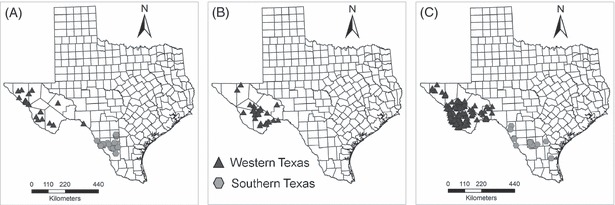
Sampling distribution of *Puma concolor* throughout western and southern Texas, USA. (A) Samples from western Texas during 1935–1955 (median = 1938; *n* = 27) and southern Texas during 1934–1942 (median = 1937; *n* = 34). (B) Samples from western Texas during 1979–1989 (median = 1983; *n* = 42). (C) Samples from western Texas during 2000–2010 (median = 2006; *n* = 168) and southern Texas during 1985–2009 (median = 1996; *n* = 28).

Mountain lions in Texas, USA, represent the eastern periphery of the contiguous distribution; breeding populations occur only in the western and southern portions of the state (Fig. 1; Schmidly 2004). The harvest of mountain lions is not regulated in Texas and mandatory inspection is not required (Harveson et al. 1996; Anderson et al. 2010). Therefore, harvest cannot be used by managers to inform demographic indices of population trends (e.g. Anderson and Lindzey 2005). Furthermore, little information is available to assist mountain lion management in Texas. Previous studies indicate Texas populations have young age structures (Harveson et al. 1996) and exhibit low survival (Harveson 1997; Young et al. 2010) and reproduction (Harveson 1997; Pittman et al. 2000). Genetic data suggest low diversity in southern Texas and genetic differentiation between southern and western Texas, implying southern Texas may be isolated (Walker et al. 2000). However, it remains unclear whether low diversity and high differentiation are a result of historical or recent events.

Unlimited harvest and sparse information warrant conservation concern for mountain lions in Texas. Thus, our goal was to assess the demographic history of mountain lions over the past century using microsatellite DNA data. Microsatellite loci are highly variable genetic markers and consist of tandem repeats of a short sequence motif (Allendorf and Luikart 2007). We sampled historical and contemporary samples from western and southern Texas to examine how (i) genetic diversity, (ii) genetic differentiation and (iii) effective population size have changed over time. Our approach allowed us to determine whether high differentiation and low diversity in southern Texas (Walker et al. 2000) were a result of historical processes.

## Materials and methods

We obtained tissue samples from mountain lions collected in western and southern Texas spanning the temporal period 1905–2010. For historical samples, we collected approximately 100–200 mg of bone material taken from the maxilloturbinates of mountain lion skulls housed in museum collections, following the protocol of Wisely et al. (2004). Contemporary samples consisted of muscle tissues donated by hunters and trappers, collected from roadkills or taken from live-trapped individuals during previous research (e.g. Harveson 1997). Muscle tissue was frozen, dried or placed in lysis buffer (Longmire et al. 1997) and stored at −20°C prior to DNA extraction.

We used separate protocols to extract DNA from muscle tissue and maxilloturbinate samples. For muscle tissue, we extracted DNA using the DNeasy Tissue Kit and a commercial protocol (Qiagen, Valencia, CA, USA). For maxilloturbinates, we ground samples using a mortar and pestle and placed them in lysis buffer (0.5 m EDTA pH 8.0, 0.5% SDS and 0.5 mg/mL proteinase K; Wang et al. 2005). We handled a maximum of nine samples (eight maxilloturbinate samples and one negative control) during each extraction day to reduce the potential for cross-contamination. Samples were incubated for ≥24 h at 50°C, and we extracted DNA using a QIAquick PCR Purification Kit (Qiagen) using a modified extraction protocol developed by Wang et al. (1998) for ancient DNA samples.

Maxilloturbinates from museum specimens generally exhibit lower DNA quality and quantity than modern tissue, resulting in a higher probability of contamination during extraction and polymerase chain reaction (PCR) set-up (Wandeler et al. 2007). Therefore, we extracted DNA and prepared PCRs for maxilloturbinate samples in an isolated laboratory where no mammalian DNA had previously been extracted or amplified. Materials used for DNA extraction and PCR were designated only for that purpose and were cleaned with RNAse Away® (Molecular BioProducts, San Diego, CA, USA) or 50% bleach before and after use.

We used the PCR to amplify 10 microsatellite loci (FCA008, FCA035, FCA043, FCA077, FCA082, FCA090, FCA096, FCA133, FCA176, FCA205) described by Menotti-Raymond et al. (1999). We amplified loci individually in 10-μL reaction volumes that contained 5 μL AmpliTaq Gold® PCR Master Mix (Applied Biosystems, Foster City, CA, USA), 0.24 μm of each primer and 1–1.5 μL of extracted DNA. However, for maxilloturbinate reactions, we increased primer concentration to 0.50 μm, added 0.2 mg/μL of bovine serum albumin and increased the quantity of extracted DNA to 1.5–2.5 μL. We used a touchdown PCR profile with an initial denaturation at 94°C for 10 min, 20 cycles of 94°C for 30 s, 62°C for 30 s, 61°C for 30 s, 60°C for 30 s and 72°C for 60 s, followed by 30 cycles of 94°C for 30 s, 55°C for 90 s and 72°C for 60 s, with a final extension of 60°C for 10 min. For maxilloturbinate reactions, we reduced the first set of temperature cycles to 10 and increased the second set to 50. For each individual, we combined 3 μL of PCR product from multiple loci and applied 1.5–2 μL of the PCR product mix to a denaturing formamide (Hi-Di Formamide; Applied Biosystems) and size standard mixture (GeneScan ROX 500; Applied Biosystems). We loaded the resulting mixtures onto a 3130*xl* genetic analyser (Applied Biosystems) for fragment separation and detection. We included a positive and negative PCR control with each run through the analyser to identify contamination and ensure consistency among runs. We inspected loci and sized alleles using GeneMapper® software v4.0 (Applied Biosystems). We reanalyzed 10% of muscle tissue samples to calculate a genotyping error rate.

Additional measures are required to ensure genotypes are correct for museum samples because extracted DNA is at relatively low concentrations and quality (Wandeler et al. 2007; Casas-Marce et al. 2009). Errors can occur from contamination, allelic dropout and false alleles (Miller and Waits 2003; Wandeler et al. 2007). Therefore, in addition to the positive and negative PCR controls, we attempted to amplify extraction negatives several times to detect potential cross-contamination during DNA extraction. Finally, we performed two to five separate reactions for each individual at each locus, only called alleles we observed ≥2 times, and calculated quality indices (Miquel et al. 2006) for data used in analyses.

### Data analysis

The historical and recent samples represented three temporal periods for western Texas (1935–1955, 1979–1989 and 2000–2010) and two periods for southern Texas (1934–1942 and 1985–2009). Each temporal sample had *n* ≥ 27 individuals, and our total data set included 299 mountain lions. The median years for the western Texas samples were 1938, 1983 and 2006, and the southern Texas medians were 1937 and 1996 (see Appendix A for museum specimens used).

We created input files for data analysis using the computer program convert (Glaubitz 2004). We tested Hardy–Weinberg expectations (HWE) using *F*_IS_ (Weir and Cockerham 1984) for two pooled statewide samples spanning the temporal periods of 1934–1955 and 1985–2010. We also assessed HWE for each temporal sample (i.e. southern Texas: 1937, 1996; western Texas: 1938, 1983 and 2006). We evaluated statistical significance (two-sided) by comparing the observed *F*_IS_ value against a null value computed from 1023 permutations of alleles among individuals in the computer program arlequin 3.5 (Excoffier and Lischer 2010).

We performed several analyses to characterize genetic diversity over time. We estimated observed heterozygosity (*H*_O_), expected heterozygosity (*H*_E_; Nei 1987), number of alleles (*A*) and allelic richness (*a*_r_) per locus for each temporal sample. We calculated *H*_O_, *H*_E_ and *A* using the computer program arlequin 3.5 (Excoffier and Lischer 2010), and *a*_r_ using hp-rare 1.0 (Kalinowski 2004, 2005). We tested our hypothesis of a temporal decline in *a*_r_ within southern and western Texas using a Wilcoxon signed-rank test (one-sided). We tested *a*_r_ because during demographic declines such as population bottlenecks, alleles are lost before heterozygosity changes (Leberg 2002; Schwartz et al. 2006). Additionally, estimates of *a*_r_ use a rarefaction method (Hurlbert 1971; Kalinowski 2004) to enable comparisons among unequal sample sizes.

Previous research indicated that mountain lions in western and southern Texas may be genetically differentiated (*F*_ST_ > 0.10) and that *H*_O_ in southern Texas was 40% lower than in western Texas (Walker et al. 2000). However, inferences were limited by small sample sizes and geographical extent. We estimated *F*_ST_ (Weir and Cockerham 1984) between southern and western Texas for the temporal period of 1934–1955 and 1985–2010 to examine differentiation. Because *F*_ST_ is sensitive to levels of genetic diversity (Meirmans and Hedrick 2011), we also calculated a standardized measure of differentiation (*D*; Jost 2008) for comparative purposes. Next, we evaluated the magnitude of genetic change over time within each geographical region by calculating *F*_ST_ and *D* among the temporal samples. This analysis produced an estimate for southern Texas (1937–1996), and three estimates for western Texas (1938–1983, 1983–2006 and 1938–2006). We calculated *F*_ST_ using the computer program arlequin 3.5 (Excoffier and Lischer 2010) and determined statistical significance (two-sided) by comparing the observed value to a null value based on 1023 permutations of genotypes among groups (i.e. regions or temporal periods). We calculated *D* using the computer program smogd 1.2.5 (Crawford 2010).

We estimated variance (*N*_eV_) and inbreeding (*N*_eI_) effective population size for southern and western Texas to explicitly test for changes in population size over time. Effective population size is the size of an idealized population exhibiting the same rate of genetic change as the sampled population (Wright 1931). We used temporal changes in allele frequencies (Krimbas and Tsakas 1971; Waples 1989) and linkage disequilibrium (LD) among loci (Hill 1981) to derive estimates of *N*_eV_ and *N*_eI_, respectively. Both methodologies make simplifying assumptions (e.g. Waples 1989, 2006; Luikart et al. 2010) including population closure and no substructure.

The temporal method requires two or more temporally spaced samples of a species with nonoverlapping generations to estimate *N*_eV_. When applying temporal estimators to age-structured populations, it is important to describe how samples are pooled over time, select an appropriate generation time and identify the number of generations separating samples (Waples and Yokota 2007). For each temporal sample from southern and western Texas, we used the median year as the pooled year (described previously). We considered 6 years as a mountain lion generation because it was the mean age of adults in a neighbouring population exposed to hunting (Logan and Sweanor 2001). Our temporal samples from western and southern Texas covered a range of 4–11 mountain lion generations, which should ensure relatively unbiased and precise estimates of *N*_eV_ (Waples and Yokota 2007).

We estimated *N*_eV_ using a moment-based (Krimbas and Tsakas 1971; Nei and Tajima 1981; Pollack 1983; Waples 1989), Bayesian (Berthier et al. 2002) and pseudo-likelihood (Wang 2001) method in the computer program NeEstimator 1.3 (Peel et al. 2004) and mlne 1.0 (Wang and Whitlock 2003). We employed 1000 updates in the Bayesian framework and assumed a maximum *N*_eV_ = 500 for western and southern Texas using the Bayesian and likelihood methods (Wang 2001; Berthier et al. 2002).

Estimates of *N*_eI_ do not require temporally spaced samples (Luikart et al. 2010). We explored temporal changes in *N*_eI_ using the LD approach of Waples (2006) for each temporal sample from southern and western Texas. This produced five estimates separated by 4–11 generations, which should be sufficient to detect trends in population size (Tallmon et al. 2010). Importantly, for age-structured samples, the estimates of *N*_eI_ based on the LD method reflect the effective number of breeders (*N*_b_) that produced the cohorts present (Waples and Do 2009). Therefore, we used the computer program ldne 1.31 (Waples and Do 2008) to compute *N*_b_ estimates and calculate 95% CIs following a jackknifing procedure. We employed the random mating model rather than the monogamy model because mountain lions exhibit a polygynous mating system (Murphy 1998). To reduce potential bias in the *N*_b_ estimates, we only used alleles that were present at frequencies >0.02 in analyses (Waples and Do 2009).

## Results

We genotyped 10 microsatellite loci for 299 mountain lions (2% missing data) collected from Texas (50% males, 46% females and 4% unknown). Sample sizes for the median year groups from western Texas included *n* = 27 (1938), *n* = 42 (1983) and *n* = 168 (2006), and *n* = 34 (1937) and *n* = 28 (1996) from southern Texas (Fig. 1). Positive PCR controls were consistent, and extraction and PCR negatives exhibited no evidence of contamination. Our genotyping error rate for muscle tissues was <1%. Our global quality index for maxilloturbinate samples was 0.95 and ranged from 0.73 to 1.00 per genotype.

### Genetic diversity and differentiation

We observed a statistically positive *F*_IS_ for the recent (1985–2010) statewide sample indicating a deviation from HWE (*F*_IS_ = 0.04, *P* = 0.04). The historical statewide sample (1934–1955) also exhibited a positive *F*_IS_, but was not statistically significant (*F*_IS_ = 0.05, *P* = 0.12). Both temporal groups from southern Texas (1937: *F*_IS_ = 0.04, *P* = 0.38; 1996: *F*_IS_ = 0.04, *P* = 0.54) and groups from western Texas (1938: *F*_IS_ = 0.02, *P* = 0.72; 1983: *F*_IS_ = −0.05, *P* = 0.16; 2006: *F*_IS_ = 0.00, *P* = 0.84) satisfied HWE. The departure from HWE in the recent statewide sample may be due to a Wahlund effect, which occurs when populations with differing allele frequencies are combined (Allendorf and Luikart 2007).

Estimates of *H*_O_, *H*_E_ and *A* for each temporal period in western Texas indicated only minor changes over time (Table 1), with mean *H*_E_ ranging from 0.59 to 0.56 during 1938–2006. We detected no difference in *a*_r_ (Table 1) for any comparisons within western Texas (1938–1983, 1983–2006 and 1938–2006: Wilcoxon *T* = −0.36, *P* = 0.36). Genetic diversity was similar in the historical samples from southern and western Texas. However, mean estimates of *H*_O_, *H*_E_ and *A* were comparatively low in the recent sample from southern Texas. We also observed evidence for a 9% temporal reduction in *a*_r_ (1937–1996: Wilcoxon *T* = −1.58, *P* = 0.06; Table 1).

**Table 1:** Estimates of genetic diversity (*H*_O_, *H*_E_, *A*, *a*_*r*_) per locus for geographical and temporal samples of *Puma concolor* in Texas, USA

	Western Texas												Southern Texas							
		
	1938 (*n* = 27)				1983 (*n* = 42)				2006 (*n* = 168)				1937 (*n* = 34)				1996 (*n* = 28)			
					
Locus	*H*_O_	*H*_E_	*A*	*a*_*r*_	*H*_O_	*H*_E_	*A*	*a*_*r*_	*H*_O_	*H*_E_	*A*	*a*_*r*_	*H*_O_	*H*_E_	*A*	*a*_*r*_	*H*_O_	*H*_E_	*A*	*a*_*r*_
FCA008	0.07	0.07	3	2.56	0.05	0.05	2	1.75	0.00	0.00	1	1.00	0.09	0.11	2	1.98	0.00	0.00	1	1.00
FCA082	0.70	0.77	6	6.00	0.75	0.63	5	4.89	0.52	0.62	6	4.51	0.47	0.51	5	4.50	0.58	0.53	4	3.96
FCA090	0.70	0.77	6	5.94	0.76	0.77	6	5.49	0.77	0.76	7	5.21	0.65	0.75	7	6.33	0.71	0.78	6	5.94
FCA133	0.63	0.51	5	4.56	0.57	0.55	5	4.39	0.53	0.55	6	4.80	0.71	0.57	4	3.62	0.64	0.61	5	4.68
FCA176	0.44	0.57	4	4.00	0.33	0.39	4	3.94	0.41	0.40	5	4.14	0.43	0.49	3	3.00	0.11	0.10	3	2.69
FCA035	0.59	0.60	3	3.00	0.73	0.69	4	3.94	0.66	0.63	6	4.21	0.52	0.50	4	3.58	0.42	0.48	3	2.99
FCA043	0.81	0.80	5	5.00	0.64	0.67	5	4.50	0.70	0.75	6	4.57	0.67	0.75	6	5.94	0.56	0.58	6	5.47
FCA077	0.48	0.48	2	2.00	0.55	0.54	3	2.94	0.54	0.52	3	2.55	0.41	0.46	3	2.62	0.14	0.25	2	2.00
FCA096	0.43	0.67	4	4.00	0.78	0.78	5	5.00	0.76	0.78	5	5.00	0.56	0.70	5	4.58	0.56	0.56	5	4.89
FCA205	0.67	0.65	4	4.00	0.66	0.61	4	3.51	0.67	0.63	4	3.83	0.59	0.68	4	3.95	0.46	0.52	3	2.94
Mean	0.55	0.59	4.20	4.11	0.58	0.57	4.30	4.03	0.56	0.56	4.90	3.98	0.51	0.55	4.30	4.01	0.42	0.44	3.80	3.66
SD*	0.21	0.21	1.32	1.34	0.23	0.22	1.16	1.10	0.23	0.23	1.79	1.28	0.18	0.19	1.49	1.38	0.25	0.24	1.69	1.59

* Represents the standard deviation of estimates across loci.

Estimates of both *F*_ST_ and *D* were similar in our genetic differentiation analyses. Differentiation between southern and western Texas for the historical sample was moderate (1934–1955: *F*_ST_ = 0.04, *P* < 0.01; *D* = 0.05), but approximately doubled in the recent sample (1985–2010: *F*_ST_ = 0.10, *P* < 0.01; *D* = 0.10), further supporting a Wahlund effect. We observed low yet significant genetic change over time within western Texas (1938–1983: *F*_ST_ = 0.03, *P* < 0.01; *D* = 0.02; 1983–2006: *F*_ST_ = 0.01, *P* < 0.01; *D* = 0.01; 1938–2006: *F*_ST_ = 0.02, *P* < 0.01; *D* = 0.01). Southern Texas, however, displayed genetic change seven to eight times greater (1937–1996: *F*_ST_ = 0.13, *P* < 0.01; *D* = 0.08) than western Texas over a similar temporal period (i.e. 1938–2006).

### Effective population size

Estimates of *N*_eV_ produced statistically similar means within temporal periods for western and southern Texas (Table 2). There was weak support for an increase in *N*_eV_ within western Texas, as the 95% CIs for historical (1938–1983) and recent (1983–2006) did not overlap the means. The arithmetic mean across methods for each interval in western Texas were *N*_eV (1938–1983)_ = 54, *N*_eV (1983–2006)_ = 166 and *N*_eV (1938–2006)_ = 109. Estimates based on the likelihood approach of Wang (2001) were consistently yet qualitatively higher than the moments (Waples 1989) or Bayesian (Berthier et al. 2002) estimates. The temporal interval of 1983–2006 produced the most variable estimates of *N*_eV_ in western Texas, but this interval was the shortest temporal span with only four generations separating samples. The historical (1938–1983) and overall estimates (1938–2006) captured 7 and 11 generations and were more precise, reflected by narrower 95% CIs. Temporal estimates for southern Texas were precise, with an arithmetic mean of *N*_eV (1937–1996)_ = 44. The mean estimate of *N*_eV_ for southern Texas was 60% lower than *N*_eV_ in western Texas over a similar temporal period.

**Table 2:** Estimates of variance effective population size (*N*_eV_) over three temporal periods for *Puma concolor* sampled from western and southern Texas, USA. Moments (Waples 1989), Bayesian (Berthier et al. 2002) and likelihood (Wang 2001) methods were used to derive estimates and 95% confidence intervals

Geographical region	Temporal interval	*N*	Waples 1989	95% CI	Berthier et al. 2002	95% CI	Wang 2001	95% CI
Western Texas	1938–1983	27–42	48	24–94	47	30–76	67	40–125
	1983–2006	42–168	146	62–467	125	73–204	228	113–500
	1938–2006	27–168	96	52–174	90	65–124	142	91–234
Southern Texas	1937–1996	34–28	36	20–63	53	29–65	41	28–63

The LD estimates of *N*_b_ for western Texas exhibited no statistical differences among temporal samples, suggesting the population has remained stable over time (Table 3). The *N*_b_ estimates for the 1938 and 1983 temporal period were variable, but the 2006 estimate was comparatively precise. The disparity in precision may have reflected differing sample sizes, where larger samples resulted in greater precision (Tallmon et al. 2010). In southern Texas, there was weak support for a decline in *N*_b_ over time as the 95% CIs for 1937 and 1996 did not overlap the means (Table 3). Similar to *N*_eV_ results, mean *N*_b_ for southern Texas was 67–90% lower than that for western Texas over similar temporal periods.

**Table 3:** Linkage disequilibrium estimates of the effective number of breeders (*N*_b_) for temporal samples of *Puma concolor* from western and southern Texas, USA

Geographical region	Temporal sample	*n*	*N*_b_	95% CI*
Western Texas	1938	27	63	22–∞
	1983	42	68	32–544
	2006	168	91	65–134
Southern Texas	1937	34	21	12–42
	1996	28	9	4–18

* Confidence intervals were computed using a jackknifing procedure.

## Discussion

The demographic history of many species is poorly understood. Thus, a major challenge in conservation genetic studies is to determine whether contemporary levels of genetic diversity and differentiation are the result of historical or recent events. The initial genetic analyses of Texas mountain lions presented a similar challenge (Walker et al. 2000). The authors observed low genetic diversity in southern Texas and high differentiation between southern and western Texas, but were unable to evaluate alternative hypotheses without historical samples. Furthermore, small number of samples (*n* = 16 and 9 for southern and western Texas, respectively) limited the inferential power of the analyses.

Our genetic diversity estimates in recent western and southern Texas are higher than reported by Walker et al. (2000), but generally supported their findings in that southern Texas displayed less diversity. The differing values were likely due to the additional samples and different loci used in our study. The inclusion of historical samples revealed a 9% temporal decline in *a*_r_ within southern Texas. Clearly, the lower diversity in contemporary mountain lions from southern Texas is a recent phenomenon.

The levels of diversity we documented in our sample were comparable with other mountain lion populations. Estimates for western Texas and historical southern Texas were 20–50% lower than those observed in mountain lions from South America (Culver et al. 2000), but were equivalent to large and presumably healthy populations in North America (Sinclair et al. 2001; Anderson et al. 2004; McRae et al. 2005). However, our recent estimates for southern Texas were similar to peripheral populations along the coastal region of California, USA (Ernest et al. 2003).

The genetic differentiation between historical western and southern Texas was analogous to contiguous populations of mountain lions in western North America (Sinclair et al. 2001). Contemporary genetic differentiation between western and southern Texas replicated results from Walker et al. (2000) and was similar to isolated or fragmented populations in California (Ernest et al. 2003). The increase in differentiation during the last 70 years appears to be the result of allele frequency changes (i.e. genetic drift) in southern Texas (temporal *F*_ST_ = 0.13) rather than western Texas (temporal *F*_ST_ = 0.01–0.03). Low levels of differentiation between historical southern Texas and the 1983 and 2006 samples from western Texas (*F*_ST_ = 0.04–0.05) provide further support for temporal changes occurring mainly in southern Texas. Indeed, the differentiation observed recently between mountain lions in southern and western Texas (Walker et al. 2000) was not present historically.

Estimates of *N*_eV_ and *N*_b_ substantiated our genetic diversity and differentiation findings. The temporal approach (Waples 1989) revealed similar estimates of *N*_eV_ in western Texas over time, and point estimates were similar to other temporally stable populations of large carnivores (Miller and Waits 2003). Estimates of *N*_eV_ for southern Texas were much lower than those for western Texas, providing evidence for a smaller average population size in southern Texas over the sampling interval. Estimates of *N*_b_ corroborated *N*_eV_ results, indicating no population changes in western Texas, and a lower average population size in southern Texas. However, estimates of *N*_b_ also revealed a significant population decline in southern Texas. Moreover, we discovered a disparity when comparing the estimates of *N*_b_ and *N*_eV_ for southern Texas. The recent estimate of *N*_b_ was 80% lower than the average of *N*_eV_ estimates within southern Texas, and *N*_b_ was similar to a reintroduced population of brown bears (De Barbra et al. 2010). The difference between *N*_b_ and *N*_eV_ estimates is likely because *N*_eV_ reflects the harmonic mean over the sampled time period (Waples and Yokota 2007), while *N*_b_ represents the number of breeders producing the sampled cohorts (Waples and Do 2009). In the case of southern Texas, temporal *N*_eV_ could have been influenced by larger historical population sizes, whereas recent estimates of *N*_b_ may be indicative of a small effective size in the contemporary population. This hypothesis is supported by the temporal decline we observed in genetic diversity within southern Texas.

Historically, mountain lions in western and southern Texas displayed high genetic diversity and low genetic differentiation indicative of a large population. Over time, western Texas exhibited essentially no change in diversity and effective population size and showed low levels of genetic drift. However, genetic diversity and effective size decreased in southern Texas, and genetic drift was extensive. Genetic differentiation has also doubled between western and southern Texas over time. Our findings highlight that mountain lions in western Texas have remained relatively stable, but population changes and declines have clearly occurred in southern Texas.

The human footprint may be responsible for the population stability in western Texas and reductions or changes in southern Texas. First, urban development and sprawl have increased dramatically in southern Texas along the Mexico–USA border and in central Texas. The Rio Grande Valley region of southern Texas also supports vast areas of cropland on both sides of the border. Collectively, development and agriculture have reduced and fragmented habitat for mountain lions and increased the potential for auto-collisions and other mountain lion–human conflicts. Changes in habitat connectivity could be responsible for the increase in genetic differentiation in southern Texas, the most peripheral population we sampled. In contrast, much of western Texas remains rangeland with little urban development. The large geographical area in western Texas, lack of urbanization and proximity to adjacent mountain lion populations in New Mexico, USA, and Mexico may have maintained a large effective size in the region. Movement occurs among western Texas, New Mexico and probably Mexico (Holbrook 2011), thus population boundaries in western Texas likely extend beyond state borders.

Additionally, during late 1800–mid 1900, livestock production was the dominant industry in Texas (Lehmann 1969). Predator control was widely practiced to support production, and predator removals included mountain lions (Wade et al. 1984). We found no evidence of decline in population size for western Texas. However, predator control may have reduced effective size and genetic diversity in southern Texas by removing migrants traversing between southern Texas and neighbouring populations. Finally, the distribution of mountain lions contracted during the 1900s owing to habitat alteration and likely predator control, perhaps leaving the southern Texas population mostly isolated on the eastern periphery. The central–marginal hypothesis states that peripheral populations may display smaller population sizes, fewer opportunities for gene flow and greater fluctuations in population size because of geographical range shifts (Eckert et al. 2008). Geographical location has also influenced population size and genetic diversity in other species of vagile carnivores (Schwartz et al. 2003). Compared with western Texas, southern Texas exhibited lower historical effective sizes, indicating southern Texas may have exhibited peripheral characteristics by the early 1900s. Thus, the apparent population decline in southern Texas could be related to multiple processes such as population isolation, range contraction and mortality because of predator control and other interactions with humans.

### Conservation and management implications

Our results demonstrate the utility of applying a retrospective genetic approach (Schwartz et al. 2006) to evaluate the demographic history of an elusive carnivore. Although exposed to unlimited hunting and a history of land-use change and persecution, mountain lions in western Texas appear to have remained at high and stable levels. The current level of harvest may not have a large negative effect on the population. However, our analyses offer no insight into the consequences of increasing harvest in western Texas, which could easily be realized under the current nongame classification. Additionally, it is possible that genetic connectivity to adjacent populations is assisting the stability we observed in western Texas. Thus, connectivity to proximate populations should be considered when applying habitat or population manipulations. Future research examining mountain lion survival and movements in western Texas would inform questions regarding harvest mortality and interpopulation connectivity. We suggest a management plan incorporating population monitoring is needed if the persistence of mountain lions in western Texas is desired. An approach using indices such as harvest reports (Anderson and Lindzey 2005) with genetic sampling would be prudent to expand on the baseline information we have established.

Declines have occurred in genetic connectivity, genetic diversity and effective population size for mountain lions in southern Texas. In fact, the temporal decline in diversity and current effective size are outside of the ranges suggested for long-term population persistence (Soule et al. 1986). Furthermore, the decline in diversity within southern Texas was 10–15% of the overall decline observed in Florida panthers (Culver et al. 2000), a population that has displayed physical symptoms of inbreeding depression (Roelke et al. 1993). Additional loss of diversity may occur through genetic drift if mountain lions in southern Texas continue to experience high mortality and low productivity (Harveson 1997).

Management actions are likely needed if mountain lions are to be maintained in southern Texas. First, the current population size and trend in southern Texas are unknown. Population monitoring efforts are needed to estimate occupied habitat, reproductive rates, survival and population viability without management intervention. Reporting of mountain lion harvests in southern Texas would assist monitoring efforts. If current harvest is unsustainable, regulation of harvest may be needed (Young 2009). A harvest management plan would allow managers to focus harvest on areas of potential mountain lion–human conflict, while maintaining survival rates of residents and migrants at sustainable levels. Unlike the Florida panther population, southern Texas appears to exchange migrants with neighbouring populations in western Texas, New Mexico (Holbrook 2011) and perhaps Mexico. Successful reproduction by migrants would increase genetic diversity and effective population size; attributes characteristic of large and stable populations (e.g. Spong et al. 2000). Overall, it is clear that conservation programs are likely necessary to ensure the persistence, and perhaps evolutionary potential, of mountain lions in southern Texas.

This study illustrates the important role of museum collections and genetic techniques in wildlife conservation and management. Museum samples coupled with recent genetic samples allow managers to retrospectively examine evolutionary change and establish demographic baselines for data deficient or difficult to survey populations, such as Texas mountain lions. We suggest that agencies and institutions maintain or establish tissue archives to facilitate long-term monitoring. As genetic methods and analytical tools continue to advance at exceptional rates, tissue collections will become increasingly useful in natural resource conservation.

## Data archiving statement

Data for this study are available at Dryad: doi:10.5061/dryad.k5d9470m.

## Appendix A – Museum samples (maxilloturbinates) of *Puma concolor* from Texas, USA, used in analyses

Samples are organized by Texas counties and were sampled during 1934–1989. Samples were attained from the National Museum of Natural History (USNM), Texas Tech University (TTU), Sul Ross State University (SRSU), Field Museum of Natural History (FMNH), or Carnegie Museum of Natural History (CM), USA.

*Brewster County, Texas*: FMNH83479–FMNH83480, SRSU 2212, TTU35131, TTU41009–TTU41010, TTU41648–TTU41659, TTU41667, TTU41740–TTU41742, TTU49620–TTU49623, USNM261685.

*Culberson County, Texas*: TTU41660–TTU41661, USNM251600, USNM262111.

*Dimmit County, Texas*: USNM251393, USNM261616, USNM262475, USNM262698–USNM262699, USNM263859, USNM264679–USNM264680, USNM264680, USNM271676.

*Frio County, Texas*: USNM261750, USNM262108–USNM262109, USNM262130–USNM262131, USNM262186.

*Hudspeth County, Texas*: USNM261686, USNM262110, USNM263413, USNM263523, USNM263769–USNM263770, USNM264177, USNM264458, USNM264682, USNM265342, USNM271857–USNM271858, USNM272085, USNM272311, USNM273167.

*Jeff Davis County, Texas*: TTU41662–TTU41665.

*La Salle County, Texas*: USNM263858, USNM264379–USNM264380.

*Maverick County, Texas*: USNM262185.

*Pecos County, Texas*: TTU41666, TTU41668–TTU41669, USNM251599.

*Presidio County, Texas*: CM21404, CM21406, TTU41670–TTU41677, USNM263772–USNM263773, USNM271675.

*Terrell County, Texas*: SRSU 2869.

*Val Verde County, Texas*: USNM261614.

*Webb County, Texas*: USNM251375, USNM251418, USNM251468–USNM251469, USNM261615, USNM263775–USNM263776, USNM263860, USNM264178–USNM264180, USNM264678, USNM272086, USNM272310, USNM272350.
